# Evaluation of QRS duration and presence of fragmented QRS in patients with inflammatory bowel disease

**DOI:** 10.7150/ijms.95653

**Published:** 2024-05-05

**Authors:** İbrahim Ethem Güven, Mustafa Candemir, Ertugrul Kayacetin

**Affiliations:** 1 Department of Gastroenterology, Ankara City Hospital, Ankara, Turkey.; 2 Department of Gastroenterology, Ankara Yildirim Beyazit University Yenimahalle Training and Research Hospital, Ankara, Turkey.; 3 Department of Cardiology, Gazi University School of Medicine, Ankara, Turkey.; 4 Department of Gastroenterology, Ankara Yildirim Beyazit University School of Medicine, Ankara, Turkey.

**Keywords:** cardiac arrhythmia, electrocardiogram, fragmented QRS, inflammatory bowel disease, QRS duration

## Abstract

**Background:** Inflammatory Bowel Disease (IBD) is mostly characterized by gastrointestinal tract involvement, however can also be accompanied with cardiac manifestations. QRS prolongation and the presence of QRS fragmentation (fQRS) have been previously evaluated in many chronic inflammatory diseases, as an independent predictor of cardiac events. In this study, we aimed to evaluate the QRS duration and fQRS in patients with IBD.

**Methods:** The presented study was designed as a single-center retrospective cohort study. The study population consisted of 217 patients with IBD and 195 healthy controls. QRS duration and presence of fQRS were evaluated using a 12-lead electrocardiogram. These parameters were compared between groups.

**Results:** QRS duration was demonstrated to be higher in the IBD group compared to the control group (92 (86-98) vs. 82 (75-90), p<0.001). The presence of fQRS was significantly higher in the IBD group (n=101 (47%) vs n=59 (30%), p=0.006). In addition, a positive correlation was demonstrated between QRS duration and disease duration (Spearman's Rho= 0.4, p<0.001). Notably, disease and QRS duration were significantly higher in the fQRS (+) group (102 (56.5-154) vs. 55 (24.3-118.3), <0.001; 94 (86-100) vs. 92 (84-96), 0.016; respectively).

**Conclusion:** Our results demonstrated that QRS prolongation and the presence of fQRS (+) were more common in IBD patients, and associated with longer disease duration. These findings may indicate subclinical cardiac involvement in IBD. Therefore, IBD patients, especially those with long-standing disease, should be followed more closely in terms of cardiac manifestations.

## Introduction

Inflammatory Bowel Disease (IBD) is a group of disorders which is characterized by the chronic inflammation of intestinal mucosa secondary to abnormal response of the immune system 1. Ulcerative Colitis (UC) and Crohn's Disease (CD) are the two main diseases of IBD, which differ in the behavior pattern and clinical course, but both are characterized by the primary involvement and chronic inflammation of the gastrointestinal system (GIS) 2. Although IBD mainly involves GIS, the inflammation is not limited to the GIS tract and may progress with other organ systems involvement, which is defined as extraintestinal manifestation 3. Among extraintestinal manifestations, cardiac involvement requires attention as they carry significant clinical implications when left unaddressed 4.

Electrocardiogram (ECG) is the most frequently used diagnostic tool in evaluating and following of cardiovascular diseases 5. Since the relationship between the chronic inflammatory state and cardiovascular diseases has been well established in the literature, pathological changes in ECG in inflammatory systemic diseases have gained interest in recent years 6. In this concept, prolongation in QRS duration, which is related to an increased risk of arrhythmia, and the presence of fragmented QRS (fQRS), which is a sign of myocardial fibrosis has been widely studied in patients with chronic inflammatory systemic diseases 7-10. To the best of our knowledge, fQRS and QRS duration have never been studied in IBD patients before.

In the current study, we aimed to assess the significance of the QRS duration and presence of fQRS in patients with IBD.

## Patients and Methods

### Study populations

This retrospective observational research was conducted in the Gastroenterology clinic of our tertiary hospital. All eligible patients diagnosed with either CD or UC, followed between January 2022 and December 2023, were analyzed for inclusion. The following exclusion criteria were applied for all subjects before inclusion: i) History of coronary artery disease (detected by anamnesis, The International Classification of Diseases codes, ischemic findings on the ECG); ii) severe valvular disease and left ventricular dysfunction (patients with decreased ejection fraction) on echocardiographic examination; iii) history of arrhythmia and intraventricular conduction delay; iv) diabetes mellitus, hypertension, chronic kidney and liver diseases, chronic respiratory diseases, hyperthyroidism or hypothyroidism, electrolyte abnormalities; v) malignancy; vi) antiarrhythmic drug use (ß-Adrenergic blockers, calcium channel blockers, sodium channel blockers, potassium channel blockers); vii) Younger than 18 years old and pregnancy. In addition to the exclusion criteria mentioned above, patients with a poor-quality or low-resolution ECG were not included in the presented study in order to make a precise evaluation. Lastly, only patients in remission for at least one month, followed by a diagnosis of IBD for at least one year, were included in the study. Crohn's Disease Activity Index (CDAI) for CD and Partial Mayo Score for UC were used for remission criteria 11. Patients with Partial Mayo Score of <2 and CDAI score of <150 were considered to be in remission.

An age- and gender-matched control group was selected from the patients who were admitted to the outpatient clinic of our hospital with complaints of dyspepsia, and no pathology was detected. Before the patients were included in the control group, it was confirmed by anamnesis that they did not have any underlying disease and did not receive any medication. Exclusion criteria were also applied to the control group.

Ethical approval was retrieved from the Local Institutional Review Board (Approval No: E1-23-4478).

### Data collection and electrocardiographic assessment

Demographic data, including age, gender, disease duration, body mass index (BMI), CD localization, CD behavior, presence of perianal disease (P), history of prior major abdominal surgery, UC extension, and current medication, were collected through electronic and printed medical records. Laboratory data of both the IBD patients and the control group were extracted from hospital digital records. The Montreal classification was used to classify the clinical features of CD. The degree of involvement of UC is categorized as proctitis, left-sided colitis, and extensive.

12-lead ECG records of both the IBD patients and the control group were obtained from the hospital's electronic records. ECGs were downloaded in high-resolution portable document format. All ECGs were magnified 600 times during the examination process to achieve sensitive measurements. The cardiologist was blinded to the subjects' data during the evaluation process. The definition of fQRS was made by the following ECG findings: The appearance of an additional R wave (R') or notching in the nadir of the R wave or the S wave, or the presence of fragmentation (at least more than one R') in 2 contiguous leads. QRS duration was calculated by measuring the interval from the beginning of the Q wave to the end of the S wave.

### Statistical analysis

Statistical analyses were performed using SPSS software version 25.0 (IBM Corp., Armonk, N.Y., USA). The normality distribution of numerical variables was evaluated by using the Kolmogorov-Smirnov test. Numerical variables were expressed as mean ± standard deviation (SD) and were compared by using the Student's t-test. Non-normally distributed numerical variables were expressed as median (interquartile range) and were compared by using the Mann-Whitney U test. Categorical variables were given as frequency (percentages) and the Chi-square test was used to make comparisons. The correlation between disease duration and QRS interval was evaluated using Spearman's test. Variables showing normal distribution patterns were assessed by one-way analysis of variance (ANOVA) test; variables not showing normal distribution patterns were assessed by the Kruskal-Wallis test. A 2-sided P <0.05 was considered significant.

## Results

A total of 412 subjects were enrolled for the study. Of the study group, 217 (52.6%) patients were in the IBD group, and 195 patients (47.3%) were in the control group. The median age was 43 (32-53) years in the IBD group and 44 (30-55) years in the control group. The male gender ratio of the IBD patients and the control group were 46% (n=100) and 42% (n=82), respectively. The median disease duration of the IBD group was 82 (31.5-139.5) months. Baseline characteristics, laboratory data, and 12-lead ECG findings are presented in Table 1. No statistically significant difference was demonstrated regarding the laboratory data except for sedimentation rate and CRP value, which was observed statistically higher in the IBD group (13 (6-25.5) vs. 9 (4-15), p<0.001; 4.3 (1.1-14.2) vs. 3.4 (1.3-6), p=0.001; respectively).

The demographic characteristics, laboratory data, clinical features, current medication, and ECG findings of our study's IBD cohort are detailed in Table 2, which delineates the CD and UC groups. No differences were demonstrated in group comparisons in terms of laboratory data, disease duration, QRS duration and rate of fQRS.

**Figure 1:**
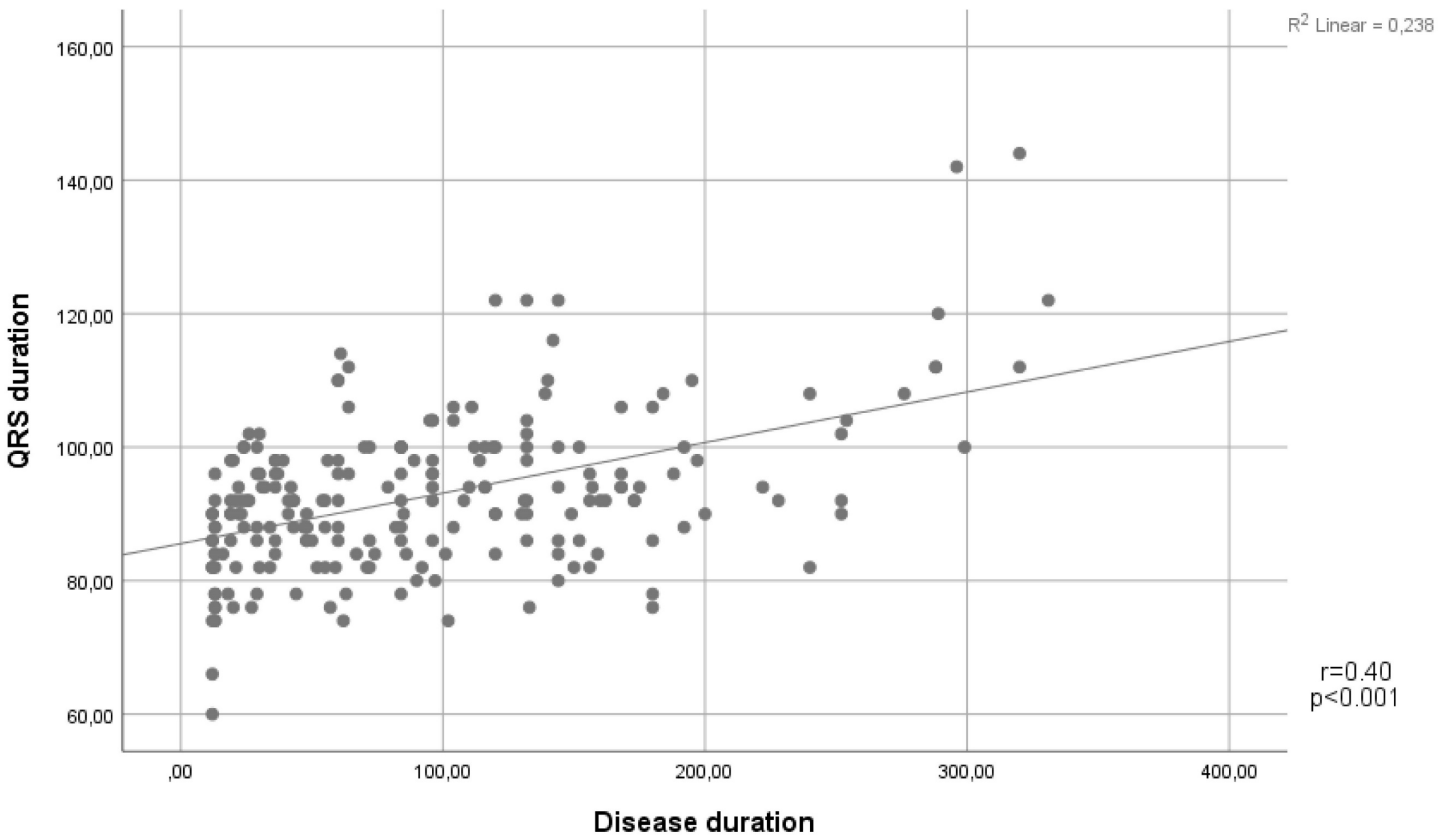
Scatter plot showing a positive linear correlation between QRS duration and disease duration.

In terms of the ECG findings, QRS duration was found to be higher in the IBD group than in the control group (92 (86-98) vs. 82 (75-90), p<0.001). In addition, the presence of fQRS was significantly higher in the IBD group (n=101 (47%) vs n=59 (30%), p=0.006) (Table 1). Notably, a positive correlation was demonstrated between QRS duration and disease duration (Spearman's Rho= 0.4, p<0.001) (Figure 1).

When IBD patients were evaluated into two separate groups, fragmented QRS (+) and fragmented QRS (-), no difference was found in group comparisons regarding demographic characteristics and laboratory data. However, higher disease duration and QRS interval were demonstrated in the fragmented QRS (+) group (102 (56.5-154) vs. 55 (24.3-118.3), <0.001; 94 (86-100) vs. 92 (84-96), 0.016; respectively) (Table 3).

As expected, we found that those with longer disease diagnosis times were older. Additionally, as the duration of the disease increased, the QRS interval lengthened, and the incidence of fQRS increased (p<0.001 for both parameters). We also found that disease duration was not related to demographic characteristics and laboratory parameters (p>0.05) (Table 4).

## Discussion

In the current study, we found that, although within the normal reference limits, QRS duration was longer, and the presence of fQRS was higher in patients with IBD. Notably, a positive correlation was demonstrated between the disease duration and the QRS duration. In addition, it was observed that the QRS duration and disease duration were higher in the fragmented QRS (+) group than in the fragmented QRS (-) group.

IBD is mainly characterized by gastrointestinal symptoms such as persistent diarrhea, rectal bleeding, and abdominal pain 12. In the past, IBD treatment and management focused intensively on the GIS system 13. However, today, with the increase in knowledge and awareness about IBD, extraintestinal system involvement has also gained importance 14. Offered data showed that inflammation secondary to IBD is not limited to the GIS tract, and extraintestinal manifestations can also be seen in its course, including musculoskeletal, cutaneous, ocular, hepatobiliary, and cardiac manifestations 15. Among them, cardiac manifestations maintain their importance due to their association with increased mortality and morbidity when left unaddressed 4. The cardiac manifestations comprise myocarditis, pericarditis, venous and arterial thrombotic events, atherosclerotic cardiovascular disease, arrhythmias, and conduction disorders 16.

The underlying mechanism of cardiac manifestations is mainly due to pathophysiological changes secondary to the chronic inflammatory state 17. Increased pro-inflammatory cytokines provoked by inflammation promote atherogenesis and endothelial dysfunction 18. An increase in pro-inflammatory cytokines plays a key role in the development of endothelial dysfunction by causing a decrease in nitric oxide release and an increase in endothelin and procoagulant factor levels. Furthermore, increased GIS permeability and increased GIS microbiate derived metabolites induced by these cytokines also constitute a risk factor for the development of endothelial dysfunction 19. In addition, exacerbated oxidative stress also contributes to these pathological changes 20. Moreover, this cumulative damage leads to fibrinogenic remodeling in the cardiomyocytes 21. Lastly, the fibrogenic mechanism negatively affects cardiac functions and heightens the risk of arrhythmia since the cardiac conduction system is also affected by fibrosis 22.

QRS duration represents ventricular depolarization and prolongation in QRS duration was found to be associated with an increased risk of arrhythmia and cardiovascular events 23. In a retrospective multicenter study, Hathaway et al. reported that prolonged QRS duration has prognostic significance in predicting mortality in patients with myocardial infarction 24. Similarly, in a prospective study by Jimenez-Candil et al., prolongation in QRS duration was demonstrated to be associated with an increased risk of cardiovascular mortality in patients with acute coronary syndrome 25. In addition, several studies demonstrated the relationship between QRS prolongation and increased ventricular arrhythmia risk 26. According to the results of this study, it is noteworthy that the QRS duration was longer in IBD patients than in the healthy control group. Furthermore, the prolongation of QRS with increasing disease duration suggests that the conduction system, which is more exposed to chronic inflammatory stare, may be subclinically impaired. Notably, QRS duration has been shown to be related to disease duration in many systemic inflammatory diseases 27,28. In our study, it was not surprising to find that QRS duration was longer in the fQRS (+) group, since fQRS (+) group has a longer disease duration. However, in the literature, this situation also can be explained as either fragmentation on the QRS complex induced by prolongation in QRS time or fragmentation on the QRS causing an increase in the duration of the QRS complex 29. Since QRS prolongation may be related to an increased risk of arrhythmia, QRS prolongation in IBD patients is an issue that needs to be discussed in further studies.

Myocardial fibrosis is one of the other consequences of fibrinogenic remodeling facilitated by chronic inflammation 30. Offered data showed that fragmentation of QRS is an indicator of subclinical myocardial fibrosis and is associated with poor outcomes 31. The significance of fQRS was evaluated in previous studies, especially in patients with chronic inflammatory disorders. In studies conducted in chronic inflammatory diseases such as Behçet's disease, systemic lupus erythematosus, ankylosing spondylitis, and rheumatoid arthritis, the presence of fQRS was found at higher rates compared to the control group 10, 32-34. In accordance with the previous studies, our study also found a higher rate of fQRS in IBD patients than in the control group. Furthermore, when the fQRS (+) and fQRS (-) groups were compared with each other, it was found that the disease duration was longer in the fQRS (+) group. In addition, when the patient groups were compared by quartile of disease duration, an increase in both QRS duration and the incidence of fQRS was observed with increasing disease duration. Since IBD is a chronic disorder, these findings may suggest the impact of cumulative stress on the cardiac system caused by the chronic inflammatory state and associated pathophysiological remodeling. However, although these findings have been shown to be associated with cardiovascular adverse events, it cannot be concluded that every case with these findings will develop these adverse events in the future clinical course.

The major limitation of the current study was that clinical follow-up of the participants could not be performed due to its retrospective design to detect adverse cardiac outcomes that may develop in the future clinical course. Secondly, the possible effects of drug groups, such as immunosuppressants and biological agents used in the treatment of IBD, on our results could not be provided. Thirdly, the study was a single-center study and had a relatively small sample size. Lastly, since our study was retrospective, case-control matching is prone to biases. Therefore, prospective studies with long-term clinical follow-up of patients are warranted.

In conclusion, we demonstrated that QRS duration and presence of fQRS were higher in patients with IBD and positively correlated with disease duration. ECG remains important in the evaluation of cardiac diseases as an easily accessible, inexpensive, and non-invasive test. Identification of risk factors for cardiac manifestations that can be detected on ECG can contribute to improving the long-term clinical outcomes of patients with IBD. Considering the increased risk of cardiovascular disease in IBD patients, clinicians may evaluate patients for further investigation in the presence of these disrupted ECG findings, particularly patients with long-standing disease.

## Author Contributions

İ.E.G. and E.K. conceived and designed the study. İ.E.G. and M.C. collected and analyzed the data, drafted the manuscript and revised it. E.K. supervised the study and revised the manuscript critically for important intellectual content. All the authors read and approved the final version of the manuscript.

## Figures and Tables

**Table 1:** Baseline characteristics, laboratory, and 12-lead Electrocardiographic parameters of the study participants.

	IBD (n=217)	Control (n=195)	*P*
Age, years	43 (32-53)	44 (30-55)	0.675
Sex (Male), n (%)	100 (46)	82 (42)	0.411
Smoking, n (%)	46 (21)	50 (26)	0.287
BMI, kg/m^2^	25 (23.1-26.5)	25 (23.2-27)	0.280
WBC, ×10^3^/ml	7.3 (5.7-8.6)	7.2 (6.2-8.5)	0.744
Hemoglobin, g/dL	13.4 (12-14.8)	13.9 (12.4-14.7)	0.152
Creatinine, mg/dL	0.8 (0.6-0.9)	0.8 (0.6-0.9)	0.207
Sodium, mmol/L	140 (139-141)	140 (137-142)	0.212
Potassium, mmol/L	4.2 (4-4.5)	4.2 (3.9-4.5)	0.350
Calcium, mg/dL	9.6 (9.2-9.9)	9.5 (9-9.9)	0.187
Total cholesterol, mg/dL	167 (143-194)	167 (138-197)	0.944
Triglyceride, mg/dL	125 (86.5-166.5)	111 (73-167)	0.214
HDL, mg/dL	45 (37-55)	45 (38-52)	0.656
LDL, mg/dL	90 (72.5-115)	96 (69-121)	0.605
Sedimentation rate, mm/h	13 (6-25.5)	9 (4-15)	**<0.001**
C-reactive protein, mg/dL	4.3 (1.1-14.2)	3.4 (1.3-6)	**0.001**
Heart rate, beat/min	78 (69-88)	77 (71-85)	0.673
QRS interval, ms	92 (86-98)	82 (75-90)	**<0.001**
QTc interval, ms	424 (408-438)	435 (376.7-495.7)	0.075
fQRS, n (%)	101 (47)	59 (30)	**0.001**
Disease duration, months	82 (31.5-139.5)	-	-

Results are expressed as mean ± SD or median (IQR) or frequency (%). SD: Standard deviation, IQR: Interquartile range, IBD: inflammatory bowel disease, BMI: body mass index, WBC: white blood cell, HDL: high-density lipoprotein, LDL: low-density lipoprotein, QTc: Corrected QT interval, fQRS: Fragmented QRS. Statistically significant results (p<0.05) were shown in bold type.

**Table 2:** Baseline characteristics, laboratory and 12-lead Electrocardiographic parameters of the patients with inflammatory bowel disease.

	Ulcerative colitis (n=78)	Crohn (n=139)	*P*
Age, years	40.5 ± 14.2	41.2 ± 12.6	0.745
Sex (Male), n (%)	35 (45)	65 (46.8)	0.789
Smoking, n (%)	16 (20.5)	30 (21.6)	0.853
BMI, kg/m^2^	25 (23.3-26)	25 (23-26.2)	0.896
WBC, ×10^3^/ml	7.4 ± 2.2	7.5 ± 2.3	0.642
Hemoglobin, g/dL	12.8 (11.9-14.4)	13.4 ± 1.8	0.290
Creatine, mg/dL	0.76 ± 0.2	0.77 ± 0.2	0.932
Sodium, mmol/L	140 (139-141.3)	140 (139-141)	0.729
Potassium, mmol/L	4.3 (4.1-4.6)	4.2 (4-4.4)	0.340
Calcium, mg/dL	9.5 (9.2-9.8)	9.6 (9.2-9.9)	0.061
Sedimentation rate, mm/h	15 (6-23.2)	13 (6-27)	0.960
C-reactive protein, mg/L	3.4 (1.4-11.0)	5.3 (1-16)	0.569
Prior major abdominal surgery, n (%)	7 (9)	38 (27.3)	**0.001**
Current medication			
*Steroids*	2 (2.6)	10 (7.2)	0.152
*5-ASA*	74 (94.9)	46 (33.1)	**<0.001**
*Immunsuppressant*	12 (15.4)	51 (36.7)	**0.001**
*Biologics*	30 (38.5)	100 (71.9)	**<0.001**
Disease duration, months	93.3 ± 75.4	95.4 ± 74.1	0.840
Heart rate, beat/min	78.4 ± 13.3	80.3 ± 14.4	0.327
QRS interval, ms	92 (84-98)	92 (86-100)	0.647
QTc interval, ms	424.6 ± 22.8	423.1 ± 22.7	0.627
fQRS, n (%)	33 (42.3)	68 (48.9)	0.349
UC Extension			
Proctitis	41 (52.6)	-	-
Left-sided	8 (10.3)	-	-
Extensive	28 (35.9)	-	-
Partial Mayo score	0 (0-1)	-	-
CD Localization			
*Ileal (L1)*	-	87 (62.6)	-
*Colonic (L2)*	-	9 (6.5)	-
*Ileocolonic (L3)*	-	43 (30.9)	-
CD Behavior			
*Inflammatory disease (B1)*	-	96 (69.1)	-
*Stenosing (B2)*	-	26 (18.7)	-
*Penetrating (B3)*	-	17 (1.2)	-
Perianal Disease	-	32 (23)	-
CDAI score	-	49.9 ± 15.8	-

Results are expressed as mean ± SD or median (IQR) or frequency (%). SD: Standard deviation, IQR: Interquartile range. BMI: body mass index, WBC: white blood cell, 5-ASA: mesalazin, UC: ulcerative colitis, CD: crohn disease, CDAI: crohn disease activity index, QTc: Corrected, fQRS: Fragmented QRS.

**Table 3:** Baseline characteristics, laboratory, and 12-lead Electrocardiographic parameters of IBD patients in the fQRS (+) and fQRS (-) subgroups.

	Fragmented QRS (+) (n=101)	Fragmented QRS (-) (n=116)	*P*
Age, years	43.3 ± 12.8	42.2 ± 13.5	0.691
Sex (Male), n (%)	50 (50)	50 (43)	0.345
Smoking, n (%)	22 (22)	24 (21)	0.844
BMI, kg/m^2^	25 (24-26.2)	25 (23-26)	0.446
WBC, ×10^3^/ml	7.3 (5.8-8.5)	7.5 (5.7-8.9)	0.509
Hemoglobin, g/dL	13.6 (12.5-15.2)	13 (11.8-14.6)	0.065
Creatinine, mg/dL	0.7 (0.6-0.9)	0.8 (0.6-0.9)	0.602
Sodium, mmol/L	140 (139-141)	140 (138-141)	0.554
Potassium, mmol/L	4.3 (4-4.5)	4.2 (4-4.5)	0.427
Calcium, mg/dL	9.6 (9.3-9.9)	9.5 (9.2-9.8)	0.097
Sedimentation rate, mm/h	12 (6-26.5)	14 (6-24.8)	0.790
C-reactive protein, mg/dL	3.3 (1-12)	5.9 (1.4-15.8)	0.179
Disease duration, months	102 (56.5-154)	55 (24.3-118.3)	**<0.001**
Heart rate, beat/min	80 ± 12.9	79.3 ± 14.8	0.555
QRS interval, ms	94 (86-100)	92 (84-96)	**0.016**
QTc interval, ms	425 (405.5-428)	424 (411-429.5)	0.718

Results are expressed as mean ± SD or median (IQR) or frequency (%). SD: Standard deviation, IQR: Interquartile range, IBD: inflammatory bowel disease, BMI: body mass index, WBC: white blood cell, QTc: Corrected QT interval, fQRS: Fragmented QRS. Statistically significant results (p<0.05) were shown in bold type.

**Table 4:** Baseline characteristics, laboratory, and 12-Lead Electrocardiographic parameters of disease duration quartile.

	Quartile 1 12-31months (n=54)	Quartile 2 32-82 months (n=55)	Quartile 3 83-140 months (n=54)	Quartile 4 141-331 months (n=54)	*P*
Age, years	38.8 ± 13.2^a^	37.3 ± 13.5^a^	45.9 ± 12.1^b^	48.6 ± 10.3^b^	**<0.001**
Sex (Male), n (%)	19 (35.2)	31 (56.4)	26 (48.1)	24 (44.4)	0.167
Smoking, n (%)	11 (20.4)	15 (27.3)	8 (14.8)	12 (22.2)	0.460
BMI, kg/m^2^	25 (22.7-26.6)	25 (23-26.3)	25 (23-26.6)	24.5 (24-25.7)	0.906
WBC, ×10^3^/ml	7.2 (5.6-8.9)	7.2 (5.7-8.8)	7.5 (6.1-8.4)	7.5 (5.4-8.8)	1.000
Hemoglobin, g/dL	13.9 (11.8-15)	13.1 (12.2-15)	13.6 (12.1-14.9)	12.7 (11.9-14.4)	0.637
Creatinine, mg/dL	0.8 (0.6-1)	0.8 (0.6-0.9)	0.8 (0.6-0.9)	0.8 (0.7-0.9)	0.514
Sodium, mmol/L	140 (138-141)	140 (139-141)	140 (139-141.3)	140 (139-142)	0.513
Potassium, mmol/L	4.3 (4.1-4.5)	4.2 (4.0-4.5)	4.2 (4-4.4)	4.3 (4-4.5)	0.883
Calcium, mg/dL	9.6 (9.1-9.9)	9.5 (9.2-9.7)	9.6 (9.2-9.8)	9.6 (9.2-9.9)	0.745
Sedimentation rate, mm/h	8 (5-23)	14 (5-25)	13 (6.8-25.3)	16.5 (8-28.3)	0.168
C-reactive protein, mg/dL	4.5 (0.8-14.1)	3.1 (1-14.6)	4.2 (1.2-19.3)	5.5 (1.5-12.3)	0.873
Heart rate, beat/min	78.2 ± 13.2	80.6 ± 16.2	78.2 ± 12.4	81.2 ± 13.7	0.575
QRS interval, ms	88 (81-92.5)^a^	90 (86-96)^a,b^	94 (88-100)^b,c^	94 (90-108)^c^	**<0.001**
QTc interval, ms	425 (405-448)	425 (405-438)	419 (410.8-433)	425 (408-438)	0.678
fQRS, n (%)	14 (25.9)^a^	22 (40)^a,b^	33 (61.1)^b^	32 (59.3)^b^	**<0.001**

Results are expressed as mean ± SD or median (IQR) or frequency (%). SD: Standard deviation, IQR: Interquartile range, IBD: inflammatory bowel disease, BMI: body mass index, WBC: white blood cell, QTc: Corrected QT interval, fQRS: Fragmented QRS. ^a,b,c^ Denotes statistically significant columns. Statistically significant results (p<0.05) were showed in bold type.

## Abbreviations

IBD: Inflammatory Bowel Disease
UC: Ulcerative Colitis
CD: Crohn's Disease
ECG: Electrocardiogram
fQRS: Fragmented QRS
CDAI: Crohn's Disease Activity Index
